# Effect of Interaction between 17β-Estradiol, 2-Methoxyestradiol and 16α-Hydroxyestrone with Chromium (VI) on Ovary Cancer Line SKOV-3: Preliminary Study

**DOI:** 10.3390/molecules25215214

**Published:** 2020-11-09

**Authors:** Ewa Sawicka, Jolanta Saczko, Joanna Roik, Julita Kulbacka, Agnieszka Piwowar

**Affiliations:** 1 Department of Toxicology, Faculty of Pharmacy, Wroclaw Medical University, Borowska 211, 50-556 Wroclaw, Poland; agnieszka.piwowar@umed.wroc.pl; 2 Department of Molecular and Cellular Biology, Faculty of Pharmacy, Wroclaw Medical University, Borowska 211A, 50-556 Wroclaw, Poland; jolanta.saczko@umed.wroc.pl (J.S.); julita.kulbacka@umed.wroc.pl (J.K.); 3 Students’ Scientific Society at the Department of Toxicology, Faculty of Pharmacy, Wroclaw Medical University, Borowska 211, 50-556 Wroclaw, Poland; roik.joanna@gmail.com

**Keywords:** ovarian cancer, chromium (IV), 17β-estradiol, 2-methoxyestradiol, 16α-hydroxyestrone

## Abstract

Ovarian cancer is the leading cause of death from gynecologic malignancies. Some estrogens, as well as xenoestrogens, such as chromium (VI) (Cr(VI)), are indicated as important pathogenic agents. The objective of this study was to evaluate the role of estradiol and some its metabolites upon exposure to the metalloestrogen Cr(VI) in an in vitro model. The changes in cell viability of malignant ovarian cancer cells (SKOV-3 resistant to cisplatin) exposed to 17β-estradiol (E2) and its two metabolites, 2-methoxyestradiol (2-MeOE2) and 16α-hydroxyestrone (16α-OHE1), upon exposure to potassium chromate (VI) and its interactions were examined. The single and mixed models of action, during short and long times of incubation with estrogens, were applied. The different effects (synergism and antagonism) of estrogens on cell viability in the presence of Cr(VI) was observed. E2 and 16α-OHE1 caused a synergistic effect after exposure to Cr(VI). 2-MeOE2 showed an antagonistic effect on Cr(VI). The examined estrogens could be ranked according to the most protective effect or least toxicity in the order: 2-MeOE2 > E2 > 16α-OHE1. Early pre-incubation (24 h or 7 days) of cells with estrogens caused mostly an antagonistic effect—protective against the toxic action of Cr(VI). The beneficial action of estrogens on the toxic effect of Cr(VI), in the context of the risk of ovarian cancer, seems to be important and further studies are needed.

## 1. Introduction

Ovarian cancer (OCa) is the leading cause of death from gynecologic malignancies. It accounts for an estimated 239,000 new cases and 152,000 deaths worldwide annually. The epidemiological research has implicated hormonal factors in the pathogenesis of OCa [1]. Although the postulated relationship between estrogens and ovarian cancer still remains unclear, the recent epidemiological data suggest an elevated risk of ovarian cancer with estrogen only or estrogen and progesterone therapy [2,3,4]. The available experimental data demonstrated that ovarian cancer cells also have a number of estrogen-regulated pathways, similar to other hormone-dependent cancers such as breast cancer and endometrial cancer [5,6,7]. The application of hormone replacement therapy (HRT) was one of the risk factors for ovarian cancer. There occurred an increased risk of approximately 22% of OCa over 5 years in postmenopausal women using estrogens as HRT [8,9]. High levels of 17β-estradiol (E2) were often observed in ovarian cancer patients. E2 stimulated an increased proliferation of ovarian cancer cells via the inhibition of cell–cell adhesion, which promotes metastasis [10].

In spite of the available research on endogenous and exogenous estrogens in relation to gynecological cancers, little is known regarding the relationship between estrogen metabolism and the risk of ovarian cancer [4]. The available data indicate that biotransformation of E2 may regulate cell proliferation by different ways. Recently, there is an increasing interest in the role of various estrogen metabolites that may affect the risk of OCa. A dysregulated metabolism of estrogen may cause DNA damage by the formation of mutagenic DNA adducts and generation of free radicals from the metabolic activation to reactive catechol estrogens [9]. Two main pathways are characteristic for estrogen metabolism in humans: hydroxylation of the A-ring (2- or 4-hydroxy derivatives) or hydroxylation of the D-ring (at 16α position) [11]. Estradiol is hydroxylated by different cytochrome P450 (CYP) enzymes in the C-2 position to form 2-hydroxyestradiol/2-hydroxyestrone, in the C-4 position to form 4-hydroxyestradiol/4-hydroxyestrone or in the C-16 position to form estriol (16-hydroxyestradiol/16-hydroxyestrone) [12]. 2-Hydroxyestradiol is preferentially converted to 2-methoxyestradiol (2-MeOE2), which has anti-angiogenic, pro-apoptotic and antiproliferative properties [13,14,15,16,17], while 16-hydroxyestrone (16α-OHE1) is dangerous because of its potential carcinogenic properties [18]. However, no studies have been carried out regarding 16α-OHE1 in ovarian cancer. Some authors concluded that the potential role of estrogens metabolism in ovary cancer, however, is less clear [4].

Statistical analyses show, that the incidence of OCa is much higher in industrial, than in developing countries. This can be attributed to the presence of endocrine-disrupting factors (EDCs), known as xenoestrogens, in occupational and environmental exposures. Many chemical environmental compounds act like estrogens. There are natural substances as well as synthetic ones. It has been noted that EDCs can increase the risk of cancer incidence, especially hormone-dependent cancers [19]. Numerous xenobiotics act as inductors or inhibitors of different enzymes, including those involved in estrogens metabolism [20]. The estrogenic activity is also characterized for some metal ions (e.g.**,** chromium); however, the data associated with the effect of EDCs on ovarian cancer are scarce. Park et al. [19] showed that EDC-induced ovarian cancer cell growth is mediated by estrogen receptors (ERs). In addition, estrogen response element (ERE) activation is involved in EDC-induced ER activity, suggesting that xenoestrogens may induce cell proliferation.

Chromium is widely present in the environment. Its toxicity depends on its valence. Chromium (VI) (Cr(VI)) is classified by the International Agency for Research on Cancer (IARC) as a proven human carcinogen. It is easily reduced to Cr(III) and during these transformations, reactive oxygen species (ROS) and reactive nitrogen species (RNS), which have an indisputable role in carcinogenesis, can be formed [21,22,23]. However, chromium is recognized as a metalloestrogen; there are only a few reports in the available literature on chromium/estrogen interaction [24,25,26]. There is no information about the role of estradiol metabolites in exposure to chromium in cancerogenesis. No prior studies have examined the role of interaction between estrogens (E2 and its metabolites) and chromium on ovarian cancer; thus, we conducted here an examination of the interaction between estrogens and metalloestrogen (Cr(VI)) on the ovarian cancer line, SKOV-3. The interaction of estrogens with the carcinogenic metalloestrogen Cr(VI) requires special care because estrogen itself possesses carcinogenic activity, so the synergistic effect with this metalloestrogen could be very dangerous for humans. The exposure to metal ions is widespread and the elucidation of their roles in the etiology and development of hormone-related cancers may have significant implications in risk reduction and disease prevention [27].

The aim of our study was to evaluate the role of E2 and its metabolites, 2-MeOE2 and 16α-OHE1, upon exposure to the environmental toxin Cr(VI) in an in vitro model. The interactions of potassium chromate (VI) with estrogens on cell viability in vitro in malignant ovarian cancer cells (SKOV-3 resistant to cisplatin) were examined. The ultimate goal of this study was to determine the type of interaction and whether estrogens play a detoxifying or advert, toxically synergistic role under such exposure conditions. In addition, the importance of long pre-incubation of SKOV-3 cells for 7 days, subsequently exposed to toxic Cr(VI), was assessed.

## 2. Results

### 2.1. Cytotoxicity Evaluation by MTT Assay

**Figure 1:**
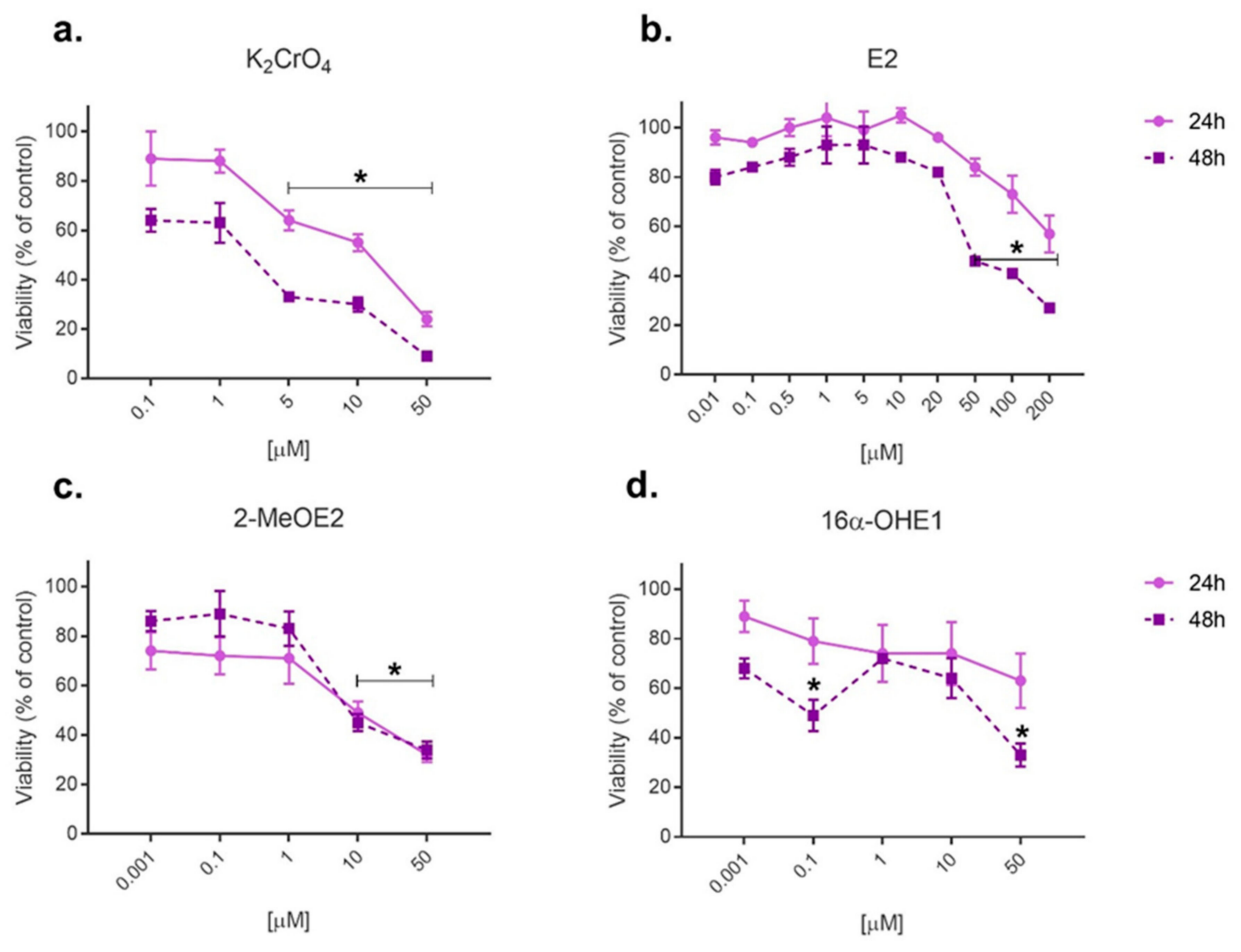
Changes in SKOV-3 viability after exposure to (**a**) K_2_CrO_4_, (**b**) 17β-estradiol (E2), (**c**) 2-methoxyestradiol (2-MeOE2) and (**d**) 16α-hydroxyestrone (16α-OHE1), measured by MTT, after 24 h and 48 h (* statistically significant, *p* < 0.05).

The investigation of single compounds on SKOV-3 cell viability was performed to establish the cytotoxicity profile of the test compounds, optimize the concentrations of estrogens and K_2_CrO_4_ and assess the combined effect of these substances. The MTT (3-(4,5-Dimethylthiazol-2-yl)-2,5-Diphenyltetrazolium Bromide) test was performed after 24 h and 48 h. Figure 1 presents SKOV-3 cell viability after Cr(VI), E2, 2-MeOE2 and 16α-OHE1 were applied.

Five doses of potassium chromate (VI) (0.1, 1, 5, 10 and 50 µM) were used. A statistically significant decrease in SKOV-3 viability was observed for Cr(VI) doses 5, 10 and 50 µM and for both incubation times—24 h and 48 h (*p* < 0.05) (Figure 1a). E2, used in high doses (0.01–200 µM), was cytotoxic for SKOV-3 cells, especially after 48 h of incubation (46% and 27% decrease in viability at the highest doses 100 and 200 µM, respectively) (*p* < 0.05). A statistically significant decrease in viability was observed for E2 concentrations at 50, 100 and 200 µM, only after 48 h of incubation (Figure 1b).

Cell incubation with high doses (10 and 50 µM) of 2-MeOE2 appeared to be more effective and comparable after 24 h and 48 h. For these 2-MeOE2 doses, SKOV-3 viability decreased to 49% and 32%, respectively, after 24 h of incubation, and to 45% and 35%, respectively, after 48 h of incubation (*p* < 0.05). For 2-MeOE2 and Cr(VI), longer incubation for 48 h resulted in a lower reduction of cell viability (Figure 1c).

The incubation with 16α-OHE1 (50 µM) decreased SKOV-3 viability after 48 h of incubation (up to 33% viability). After 24 h of incubation, the decrease in viability was proportional to the dose of the metabolite, while incubation for 48 h with 16α-OHE1 did not show such relationship. Statistically significant changes were observed only after 48 h of incubation, for two doses of 16α-OHE1 (0.1 and 50 µM) (Figure 1d).

### 2.2. The Evaluation of Simultaneous Effect of 17β-Estradiol and Its Metabolites as Well as K_2_CrO_4_—Interaction Examination

**Figure 2:**
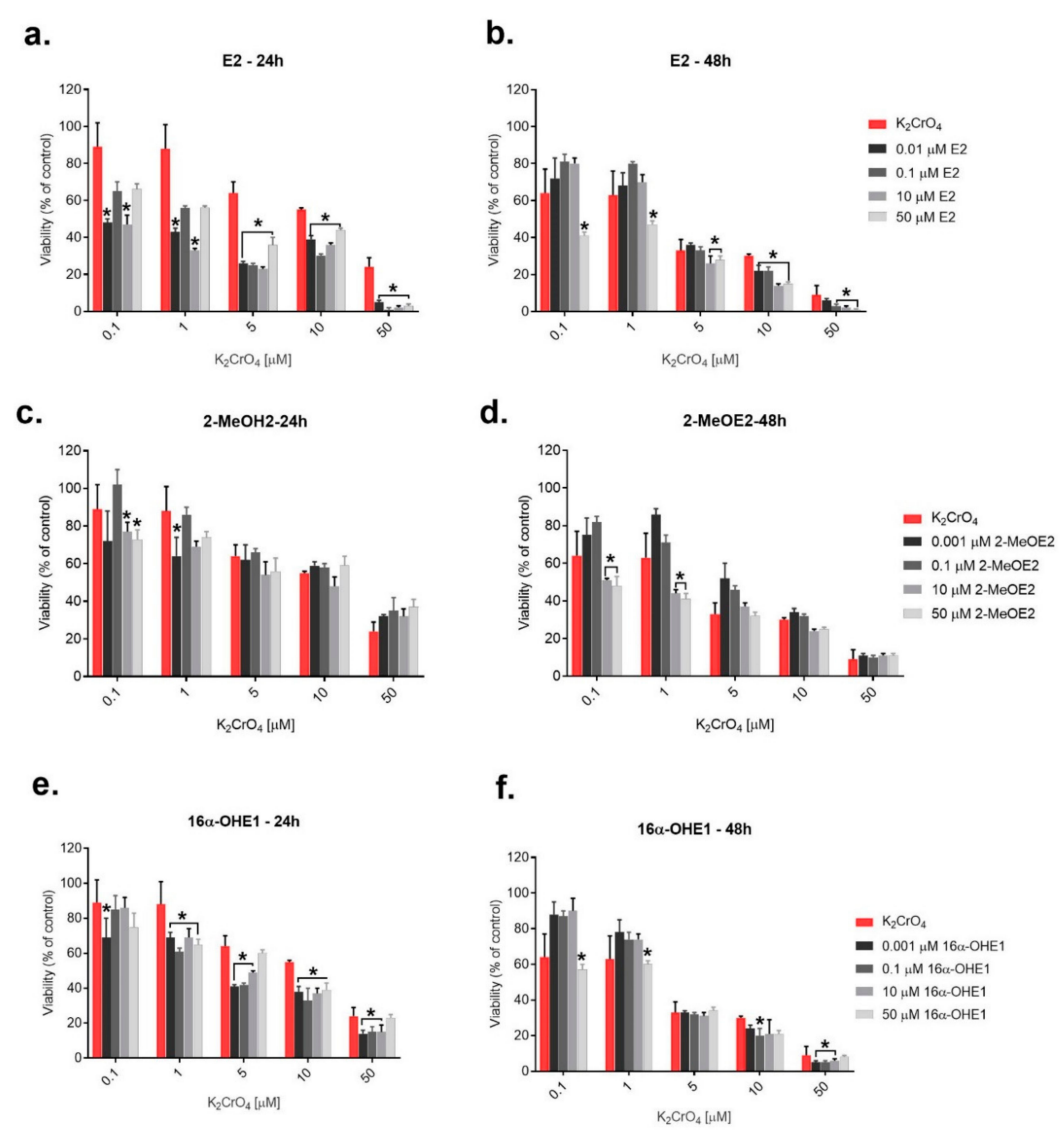
Changes in SKOV-3 viability after simultaneous effect of both E2 and K_2_CrO_4_ (**a**,**b**), 2-MeOE2 and K_2_CrO_4_ (**c**,**d**), 16α-OHE1 and K_2_CrO_4_ (**e**,**f**), measured by MTT after 24 h and 48 h (* statistically significant, *p* < 0.05).

Based on the cytotoxic profile and literature data, the abovementioned concentrations of E2 and its metabolites were chosen to study their simultaneously action with Cr(VI) on SKOV-3 viability. Simultaneously SKOV-3 incubation (24 h) of both E2 and Cr(VI) caused adverse decreasing viability in all concentrations (Figure 2a). A significant reduction of SKOV-3 viability was observed after incubation with both compounds in comparison to metalloestrogen action alone. The type of interaction between E2 and Cr(VI), after 24 h, was evaluated using the program CompuSyn. In each concentration of the tested compounds, the type of combined effect was described as synergism (Table 1). After 48 h of incubation, the SKOV-3 cell line viability increased after combined effect with E2 (0.01, 0.1 and 10 μM) in comparison to Cr(VI) alone. This effect was noted particularly for low doses of Cr(VI)—0.1 and 1 µM. The highest concentration of E2 (50 µM), in combined action with Cr(VI), decreased significantly the viability of cells in comparison to only Cr(VI) (Figure 2b) (*p* < 0.05). Antagonism was observed for the combined effect of E2 at concentrations of 0.1 and 0.01 μM with Cr(VI) in the lowest doses of 0.1 and 1 μM. For the majority of examined doses of E2 and Cr(VI), the type of interaction noted was synergism (Table 1).

The interactions of 2-MeOE2 with Cr(VI) are presented in Figure 2c,d. After incubation for 24 h, 2-MeOE2 (0.001–50 µM) alternately caused a decrease and increase in viability in comparison to Cr(VI) alone at 0.1 µM. The observed type of interaction was primarily antagonism (Table 2). Most of the different results were obtained after 48 h of incubation. Synergism and antagonism alternated, but with the predominance of antagonism (Table 2).

The combined effect of Cr(VI) (0.1–50 μM) with 16α-OHE1 (0.001–50 μM) decreased ovarian cell activity stronger than Cr(VI) alone (Figure 2e,f). The interaction of Cr(VI) at 50 μM with the metabolite decreased SKOV-3 cell viability to 14% (*p* < 0.05). In combined action, synergism was predominant (Table 3). Longer incubation for 48 h with Cr(VI) (5, 10 and 50 μM) and 16α-OHE1 caused a significant decrease in cell viability. The synergistic effect was observed for 16α-OHE1 (0.001–50 μM) and high doses of Cr(VI) (10 and 50 μM) (Figure 2f). Synergism and antagonism were alternately observed (Table 3).

### 2.3. The Evaluation of Effect of Potassium Chromate (VI) on SKOV-3 Cells after Pre-Incubation (24 h and 7 Days) with 17β-Estradiol and Its Metabolites

**Figure 3:**
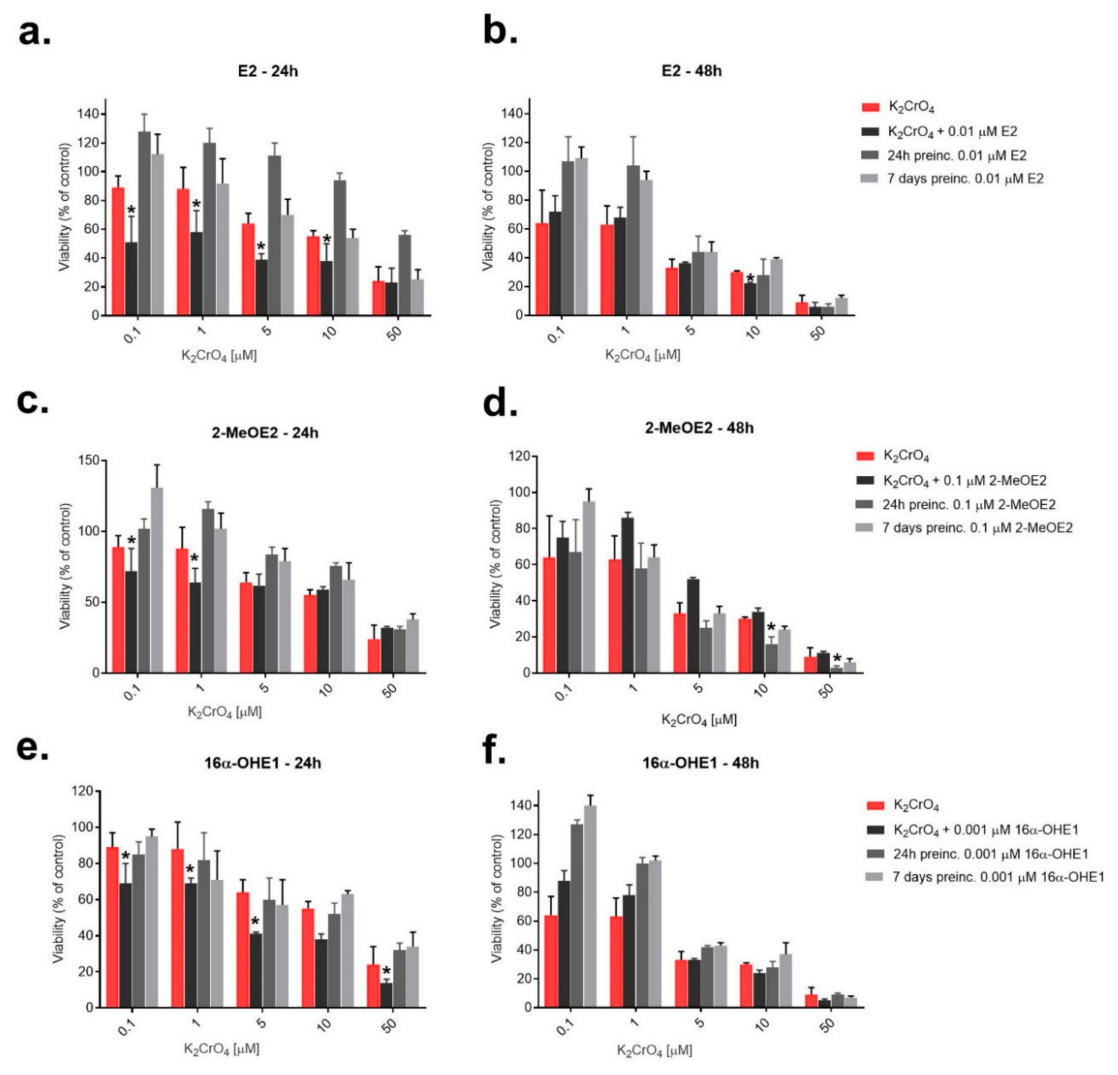
Changes in SKOV-3 viability after 24 h and 7 days of pre-incubation with E2 (**a**,**b**), 2-MeOE2 (**c**,**d**) and 16α-OHE1 (**e**,**f**), and exposure to potassium chromate (VI), measured by MTT after 24 h and 48 h. The figure also shows the combined action—the interaction of the respective estrogen with potassium chromate (VI) (* statistically significant, *p* < 0.05).

In the model of pre-incubation with estrogen, E2 or metabolites were added to cells at one appropriate concentration: 0.01 μM for E2, 0.01 μM for 2-MeOE2 and 0.001 μM for 16α-OHE1. The cells were pre-incubated in a culture bottle for 24 h or 7 days. Next, Cr(VI) at an appropriate concentration was added. The results of cell viability after 24 h and 48 h of incubation with K_2_CrO_4_ are presented in Figure 3.

After 24 h pre-incubation with E2, cell viability increased, but without statistical significance. A particular effect was observed for Cr(VI) at 50 μM, where the viability increased from 23% to 56%. Less beneficial effect was observed after pre-incubation for 7 days with E2. The combined interaction of both compounds was more toxic to cells than the action of Cr(VI) alone. The effect obtained after 48 h of incubation potentially gave protection to E2, mainly for low concentrations of Cr(VI) (0.1, 1 and 5 μM). E2 positively acted in both the pre-incubation times (24 h and 7 days), with a slight advantage of 7 days (Figure 3a,b). The results of pre-incubation of SKOV-3 cells with 0.1 µM of 2-MeOE2 during 24 h and 7 days were presented in Figure 3c,d. Both the pre-incubation times (24 h and 7 days) with 2-MeOE2 gave a protective effect against Cr(VI), which induced damage in SKOV-3 cells in all applied concentrations—the viability increased. For Cr(VI) at 0.1 μM, pre-incubation for 7 days with the metabolite was effective. At a low dose of Cr(VI) (0.1 μM), the positive effect on SKOV-3 viability was noted for pre-incubation for 7 days with 2-MeOE2.

The effects of pre-incubation of SKOV-3 with 16α-OHE1 against the toxic action of Cr(VI) were also determined (Figure 3e,f). After 24 h pre-incubation with 16α-OHE1, no significant protect outcome in SKOV-3 cells exposed to the highest Cr(VI) dose (50 μM) was observed (Figure 3e). At Cr(VI) concentrations of 0.1–10 μM, the effect of this pre-incubation was also unfavorable; the viability of cells decreased to values lower than that in the action of the metalloestrogen itself. At 0.1, 10 and 50 μM of Cr(VI), after pre-incubation for 7 days with 16α-OHE1, the viability was not significantly reduced.

Figure 3f shows the results obtained for the same compounds, but measured by the MTT test after 48 h. The toxic effect of Cr(VI) on the cells, after longer incubation for 48 h, was weakened, because 16α-OHE1 acted protectively in this case. Pre-incubation for 7 days with the metabolite positively influenced SKOV-3 cell viability. Similarly, 24 h of pre-incubation had a protective effect on SKOV-3 cells, especially for K_2_CrO_4_ at concentrations of 0.1, 1 and 5 μM. In each experiment to study the combined effect, 50% inhibition of cell growth (IC_50_ values) was also calculated. The results are presented in Tables S1–S4 (Supplementary Materials). The IC_50_ values indicated that pre-incubation with E2 and its metabolites caused a synergistic effect with Cr(VI), particularly when used with very low concentrations of estrogens.

## 3. Discussion

Epidemiological evidence intensely suggests that steroid hormones, such as estrogens and progesterone, are involved in ovarian carcinogenesis. Moreover, it seems significant to clarify the interaction between estrogens and compounds belonging to xenoestrogens, i.e.**,** substances which have the ability to interact with the estrogen receptor [3,28,29,30]. Among the wide group of xenoestrogens, metalloestrogens (e.g., chromium) seem to be important in ovarian carcinogenesis [31,32]. E2 and its metabolites, through the genomic mechanism of action, stimulate proliferation and increase the number of cell divisions. 2-MeOE2 appears to be a promising compound in the case of cancer chemotherapy, is an inhibitor of superoxide dismutase (SOD) and has the ability to damage the cytoskeleton of tumor cells or inhibit the proliferation of many cells, including cancer cells [14,28]. However, 16α-hydroxyestrone, is the most dangerous because of its potential carcinogenic properties [18]. Based on the data, the role of factors affecting the estrogen receptor and its metabolites and the development of estrogen-dependent ovarian cancer should be examined.

The present study attempted to classify the role of the steroids, E2 and its two metabolites (2-MeOE2 and 16α-OHE1), in the damage to SKOV-3 ovarian carcinoma cells exposed to hexavalent chromium. The choice of the following cell line was not accidental. We selected the SKOV-3 ovarian human cancer cell line, which expresses the estrogen receptors α and β. The estrogen receptor α (ERα) is especially expressed in more than 50% of ovarian cancer and this expression is associated with poor prognosis in ovarian cancer patients. It was interesting to check the detoxifying or toxically synergistic role of estrogen and its metabolites in cancer cells. Our preliminary study showed that 17β-estradiol and its metabolites played a dual role— detoxifying antagonistic and toxically synergistic—in SKOV-3 cells. Using other lines would not give us the opportunity to evaluate such activities. Most of the studies concerning estrogen responsiveness were performed on breast cancers. Here, we selected the SKOV-3 ovarian human cancer cell line which had not been tested in similar studies before. This cell line is resistant to the wide spectrum of cytostatic drugs (e.g., diphtheria toxin, cisplatin, and Adriamycin [33]. The obtained results from our experiments can be used in the future to design multidrug therapy, because the SKOV-3 line overexpresses ABC transporters [34,35].

However, chromium is recognized as a metalloestrogen, and there are only two reports in the available literature on the effect of chromium/estrogen interaction on oxidative stress [25,26]. These reports concern research in biological materials such as blood or isolated mitochondria, but without the use of cell lines. The application of cell cultures to study the carcinogenic mechanisms of chemical carcinogens can provide insights into the cellular and molecular mechanisms of ovarian carcinogenesis. In the first stage of our research, the effect of single hormones and potassium chromate (VI) on the cell viability of SKOV-3 was determined. After that, the combined effect of the steroid and metalloestrogen was carried out by the MTT assay in a simultaneous action setting (a kind of interaction examination), while in the third experiment, previous pre-incubation (24 h and long-lasting 7 days) with examined estrogens before exposure of SKOV-3 cells to Cr(VI) was evaluated. The cytotoxic effect of E2 on SKOV-3 cell viability was observed, especially for the highest concentrations (above 50 µM) after 48 h of incubation (*p* < 0.05). Other authors indicated that E2, even at physiological concentrations (0.01 and 10 nM) increased the proliferation of cells and decreased apoptosis in OVCAR-3 human ovarian cells [36]. Numerous data implicate a role for estrogens in ovarian carcinogenesis, although the extent of its influence and the mechanistic details are unclear. However, it has been shown that ovarian tumor cell proliferation increases with estrogen exposure [37,38,39].

Given the potential anticancerogenic role of 2-MeOE2 shown in the literature, this metabolite was examined in the present study. 2-MeOE2 in doses from 0.001 to 1 µM caused cell viability to be maintained at 80–90% in comparison to 24 h of incubation (about 70%), whereas higher doses of this metabolite (10 and 50 µM) reduced cell viability to 30–40%. The study performed by Kato et al. indicated that 2-MeOE2 alone or in combination with tumor necrosis factor (TNF) induced apoptotic cell death in ovarian cancer [39]. A similar investigation carried out by Saczko et al. showed the anticancer properties of 2-MeOE2 in connection with photodynamic therapy (PDT) in ovarian clear carcinoma cell line (OvBH-1) and activated apoptosis. [14]. Moreover, the study of Ding et al. [40] demonstrated 2-MeOE2 to be a potent inhibitor of cancer cell growth, which enhanced the effects of carboplatin in SKOV-3 cancer cells. The authors indicated on possible therapeutic applications in the treatment of ovarian cancer. The anticancer potential of 2-MeOE2 is independent on estrogen receptor binding because the affinities of estrogen receptors for 2-MeOE2 are extremely weak [14].

A number of studies suggested that under certain conditions 16α-OHE1 is involved in carcinogenesis [41,42]. A comparison of the estradiol metabolism of murine mammary epithelial cells of high-risk strains for breast cancer with those of lower risk revealed that C16-hydroxylation was significantly elevated in high-risk animals [42]. It is astonishing to note that no studies have been conducted to elucidate the biological significance of this estradiol metabolite in ovarian cancer. In our study, 16α-OHE1, particularly after 48 h of incubation, significantly decreased the viability of SKOV-3 cells, and this effect was observed mainly for the high dose (50 µM) of 16α-OHE1.

It is commonly known that hexavalent chromium induces neoplastic transformation in breast, kidney and liver cells but these properties of Cr(VI) have not been examined on ovarian cancer cells yet. This metalloestrogen may be involved in ovarian cancer development because of the abundance of estrogen receptors in the ovarian mucous membrane [22,37]. The obtained results in concentrations from 0.1 to 50 μM of Cr(VI) showed a toxic effect on SKOV-3 cells, largely after 48 h of incubation. Mostly, the high concentrations of this metalloestrogen significantly decreased SKOV-3 cell viability. So, our results revealed the cytotoxic potential of Cr(VI).

In the next step, we evaluated the combined action of E2 or its metabolites with Cr(VI) and moreover, the kind of interaction between these compounds. It was very interesting to consider whether estrogens, due to their antioxidant properties, protect the ovarian cells or lead to a toxic synergistic reaction. There is no information on the subject in the literature. The combination action of E2 and Cr(VI) after 24 h of incubation revealed the unfavorable synergistic effect of exposure to these two compounds on the SKOV-3 line. E2 did not exert a protective effect on the ovarian cell line. An analysis by the CompuSyn software also confirmed the synergistic effect of both compounds on SKOV-3 cell survival. On the other hand, longer incubation for 48 h, showed a protective role of estradiol on SKOV-3 cells, especially exposed to low doses of Cr(VI). The interaction analysis of the CompuSyn program confirmed the results obtained in the MTT test, where favorable antagonism was often observed. It can be concluded that the interaction of the metalloestrogen and E2 decreased SKOV-3 ovarian cancer cell viability and thus, this effect may be more beneficial for cancer therapy than physiological doses of the hormone.

The combined effect of 2-MeOE2 and Cr(VI) on cell viability after 24 h showed a beneficial effect on cell survival at most tested concentrations. The type of interaction (antagonism) was also confirmed in the CompuSyn software. Longer, 48 h exposure to this metabolite on chromium-induced SKOV-3 cell damage showed the protective action of 2-MeOE2 (for low concentrations of Cr(VI) and 2-MeOE2). The obtained results indicated that higher concentrations of 2-MeOE2 seemed to be advantageous from the point of view of cancer therapy when combined with hormonotherapy. Other studies showed that the anticancer activity of 2-MeOE2 is connected with damage of the cytoskeleton structure and antioxidant systems, mainly SOD system blocking [14]. It may result in increased sensitivity to oxidative factors, such as chromium compounds.

After 24 h of incubation, 16α-OHE1 did not protect SKOV-3 cells from the toxic effects of hexavalent chromium. Moreover, after 48 h of incubation, the synergistic, unfavorable, interaction effect particularly manifested after exposure to Cr(VI) in high doses (10 and 50 μM). The antagonistic effect was characteristic only for low concentrations of Cr(VI). In the case of the neoplastic process, the negative effect of this metabolite on cell survival has been observed, particularly due to the ability to generate oxygen free radicals. Some results indicate that 16α-OHE1 participates in tumor progression [32,37], but these properties have been not investigated on ovarian cancer yet.

In the last step of our observation, pre-incubation with E2 revealed positive effects on the survival of SKOV-3 cells after pre-incubation for 24 h and 7 days when exposed to Cr(VI). Our investigations proved that E2 had a protective role for cell viability when used in combination. The second pre-incubation duration of 7 days brought less beneficial results. After pre-incubation of the SKOV-3 cell line for 24 h and 7 days with 2-MeOE2, the positive outcome was observed for all concentrations of Cr(VI) because viability increased, but without statistical significance. It can be concluded that 2-MeOE2 plays a protective role against Cr(VI). However, for the low doses of Cr(VI), pre-incubation for 7 days was more protective than pre-incubation for 24 h. The toxic effect of Cr(VI) on the cells in the longer incubation period of 48 h was weakened because of the protective action of 16α-OHE1. The highest increase in cell viability was observed after pre-incubation for 7 days with 16α-OHE1. In the entire range of tested concentrations of Cr(VI), initial 24 h and 7 days of pre-incubation increased cell viability (more often observed after 48 h of incubation). The viability of ovarian cancer cells was higher after pre-incubation with the metabolite, compared with the combined effect of both compounds.

## 4. Materials and Methods

### 4.1. Cell Culture

The study was performed on one ovarian cell line (SKOV-3). The cell line was a kind gift from Professor Jakub Gołąb from the Department of Immunology, Center of Biostructure Research at Medical University of Warsaw according to our scientific cooperation. Primarily, this cell line was purchased from ATCC^®^, and validated negatively against *Mycoplasma*. Authentication was performed by a PCR-based technique that compared multiple short tandem repeat (STR) markers between two or more cellular genomes and for two months by MycoBlue (Vazyme, Lodz, Poland). The cultures were maintained in 37 °C under high humidity in the Steri-Cult automated CO_2_ incubator (Steri-Cult, Thermo Scientific, Alab, Warsaw, Poland). As the culture medium, we used Dulbecco′s Modified Eagle′s Medium (DMEM) with a high concentration of glucose (4500 mg/L) (Sigma-Aldrich, Poznan, Poland) and supplemented with 10% fetal bovine serum (FBS, Sigma-Aldrich, Poland) and 1% antibiotic solution (10,000 units penicillin and 10 mg streptomycin/mL (Sigma-Aldrich, Poznan, Poland).

### 4.2. Compounds

Four compounds were applied in the study: E2, 2-MeOE2, 16α-OHE1 and potassium chromate (VI) (K_2_CrO_4_) as a source of Cr(IV). The list of compounds is presented in Table 1. Estrogen solutions were prepared in 96% ethanol, while for K_2_CrO_4_, distilled water was used (Table 4).

### 4.3. Cytotoxicity Assay of Single Action of Estrogens and Cr(VI)

The estimation of cell viability was performed by the MTT assay according to the manufacturer protocol (Sigma-Aldrich). First, cells were incubated with estrogens (E2, 2-MeOE2 and 16α-OHE1) or Cr(VI) independently to evaluate the cytotoxicity of this compounds to SKOV-3. The following concentrations of compounds were used—E2: 0.01, 0.1, 0.5, 1, 5, 10, 25, 50, 100 and 200 µM; 2-MeOE2 and 16α-OHE1: 0.001, 0.1, 1, 10 and 50 µM; Cr(VI): 0.1, 1, 5, 10 and 50 µM. Then, some of the concentrations were chosen for further studies of combined effect of E2 or its metabolites with Cr(VI) on the SKOV-3 line. Cells were seeded onto 96-well culture plates at a concentration of 5 × 10^4^ cells/well (Nunc, Nunclon Surface, Biokom, Janki, Poland).

### 4.4. Combination Effect of Estrogen–Chromium (VI) Estimation

**Scheme 1:**
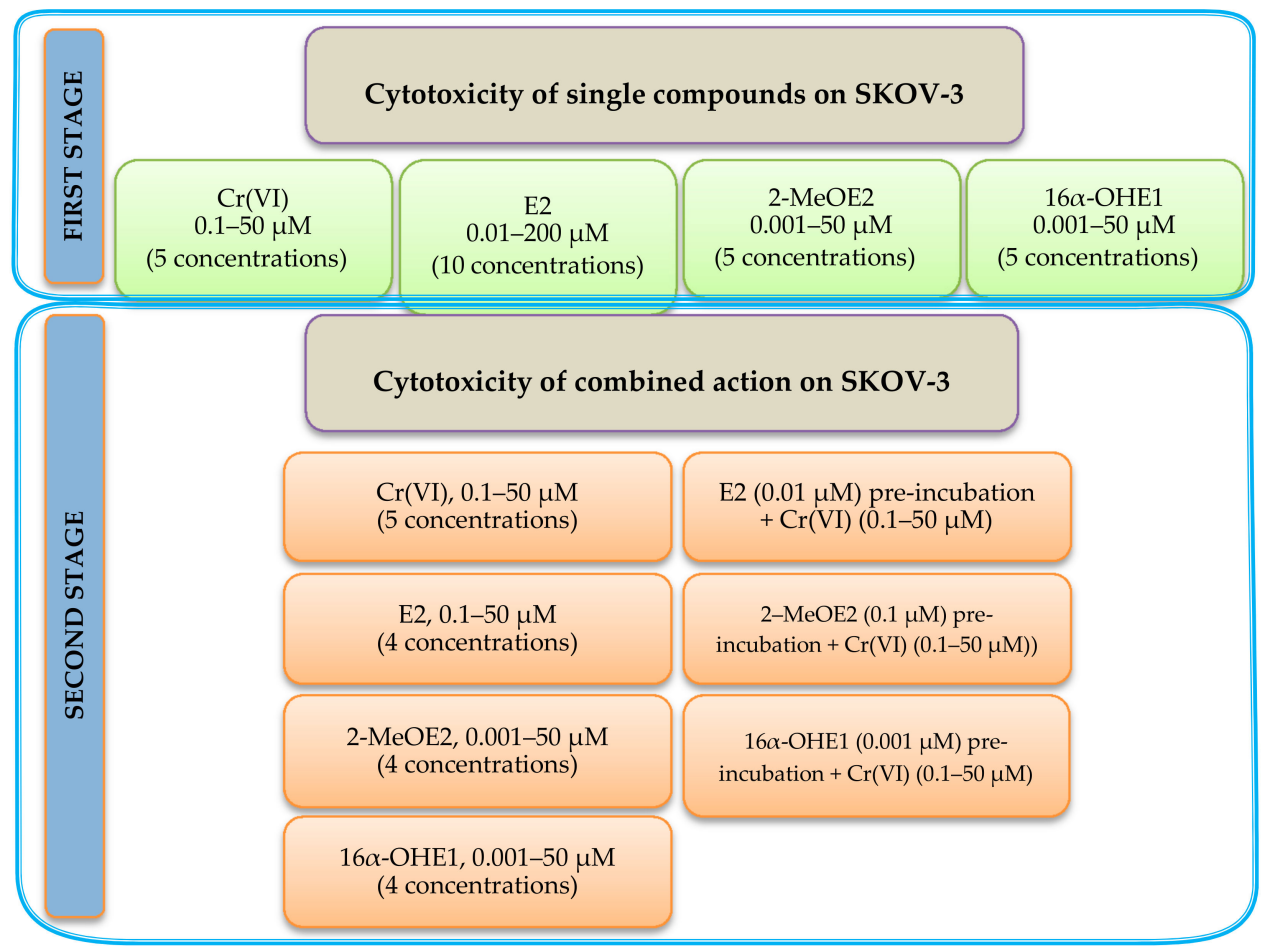
Study design for testing the cytotoxicity of 17β-estradiol and its metabolites and Cr(VI) on the SKOV-3 cell line in single and combined action.

In order to examine the combined effect, each estrogen was incubated with Cr(VI). In this first model, an estrogen and K_2_CrO_4_ were added to the cell culture simultaneously and incubated for 24 h and 48 h at 37 °C. Based on the cytotoxicity study of single compounds, the following concentrations were selected for combined effect estimation (interaction)—E2: 0.01, 0.1, 10 and 50 µM; 2-MeOE2 and 16α-OHE1: 0.001, 0.1, 10 and 50 µM. In order to evaluate the cytotoxicity of Cr(VI) on SKOV-3 in the combination effect, the following concentrations of K_2_CrO_4_ were used: 0.1, 1, 5, 10 and 50 µM (Scheme 1).

The dose effect of combination treatment with two compounds (i.e.**,** an estrogen and Cr(VI)) was evaluated using the combination index (*CI*) by the CompuSyn software. The combination index was calculated for IC_50_, obtained in an in vitro experiment. The following interpretation was used: *CI* = 1 indicates the additive effect of the test substances on the viability of ovarian cancer cells, *CI* < 1 indicates synergy between the compounds used for the study, while *CI* > 1 indicates the antagonism that occurs between them [28]. The main assumptions were calculated according to Equation (1).

(1) CIA+B=DAB+BDA+ DBA+BDB+DAA+B×DBA+BDA×DB

where *CI*(*A + B*) is the combination index for the experimentally achieved IC_50_ effect for the combination of compounds: A (estrogen) and B (chromium (VI)); *D*(*A*/*A* + *B*) is the concentration of compound A in combination A + B, resulting in IC_50_ effect; *D*(*B*/*A* + *B*) is the concentration of compound B in the combination A + B, giving the IC_50_ effect; *D*(*A*) is the concentration at which compound A exhibits an IC_50_ value; and *D*(*B*) is the concentration at which compound B exhibits an IC_50_ value.

In the second model of combination effect, cells were pre-incubated first with estrogen for 24 h and 7 days. Each estrogen was added in one selected dose: E2, 0.01 µM; 2-MeOE2, 0.1 µM; 16α-OHE1, 0.001 µM. The selection of the above doses was based on the results obtained during the estrogen–Cr(VI) interaction study (first model). Next, Cr(VI) at doses 0.1, 1, 5, 10 and 50 μM was added. The cells were incubated for 24 h and 48 h. Then, the cytotoxicity test was used. All tests were performed in triplicates.

### 4.5. Statistical Analysis

All values were expressed as mean ± SD. Differences between groups were assessed by one-way analysis of variance (ANOVA) which compares three or more unmatched groups, based on the assumption that the populations are Gaussian. The analysis was performed using the GraphPad Prism 7 software (GraphPad, DMW Communication, San Diego, CA, USA). Values of *p* < 0.05 were considered significant. The dose effect of the combination treatment was evaluated using the combination index (*CI*) by the CompuSyn software [43].

## 5. Conclusions

During our preliminary study, we observed a mainly synergistic type of interaction of chromium (VI) with estrogens. E2 and 16α-OHE1 induced a synergistic effect after SKOV-3 exposure to Cr(VI), while 2-MeOE2 showed an antagonistic effect on Cr(VI). The role of 2-MeOE2 may be a consequence of inducing oxidative stress and apoptosis in cells. Based on our research, we ranked the estrogens according to the most beneficial effect or least toxicity in the following order: 2-MeOE2 > E2 > 16α-OHE1. Moreover, it was shown that prior pre-incubation (24 h or 7 days) of SKOV-3 cells with estrogens causes an antagonistic effect against Cr(VI) toxicity in most cases. The obtained preliminary results suggest the need to continue research on the interaction of 17β-estradiol and its metabolites with metalloestrogens, in order to better understand their role in carcinogenesis and also in the context of hormone therapy for ovarian cancer.

## Figures and Tables

**Table 1:** Interpretation of the combined effect of 17β-estradiol and potassium chromate (VI) on SKOV-3 cells in the MTT test after 24 h and 48 h of incubation, using the CompuSyn program. The table summarizes the effect (**S**, synergism; **A**, antagonism).

Incubation (h)	17β-Estradiol (µM)							
Chromium (VI) (µM)	24 h				48 h			
	50	10	0.1	0.01	50	10	0.1	0.01
**50**	S	S	S	S	S	S	S	S
**10**	S	S	S	S	S	S	S	S
**5**	S	S	S	S	S	S	**A**	**A**
**1**	S	S	S	S	S	**A**	**A**	**A**
**0.1**	S	S	S	S	S	**A**	**A**	S

**Table 2:** Interpretation of the combined effect of 2-methoxyestradiol and potassium chromate (VI) on SKOV-3 cells in the MTT assay after 24 h and 48 h of incubation, using the CompuSyn program. The table summarizes the effect (**S**, synergism; **A**, antagonism).

Incubation (h)	2-Methoxyestradiol (µM)							
Chromium (VI) (µM)	24 h				48 h			
	50	10	0.1	0.001	50	10	0.1	0.001
50	**A**	**A**	**A**	**A**	S	S	S	S
10	**A**	**A**	**A**	**A**	**A**	S	**A**	**A**
5	**A**	**A**	**A**	**A**	**A**	**A**	**A**	**A**
1	**A**	**A**	**A**	S	**A**	**A**	**A**	**A**
0.1	**A**	**A**	**A**	S	**A**	**A**	**A**	**A**

**Table 3:** Interpretation of the combined effect of 16α-hydroxyestrone and potassium chromate (VI) on SKOV-3 cells in the MTT assay after 24 h and 48 h of incubation, using the CompuSyn program. The table summarizes the effect (**S**, synergism; **A**, antagonism).

Incubation (h)	16α-Hydroxyestrone (µM)							
Chromium (VI) (µM)	24 h				48 h			
	50	10	0.1	0.001	50	10	0.1	0.001
50	S	S	S	S	S	S	S	S
10	S	S	S	S	S	S	S	S
5	**A**	S	S	S	**A**	S	**A**	**A**
1	**A**	**A**	S	S	**A**	**A**	**A**	**A**
0.1	**A**	**A**	**A**	S	**A**	**A**	**A**	**A**

**Table 4:** The list of tested compounds.

The Name of the Compound	The Structure and the Manufacturer’s Name	Stock Solution	The Molecular Mass of the Compound
17β-estradiol	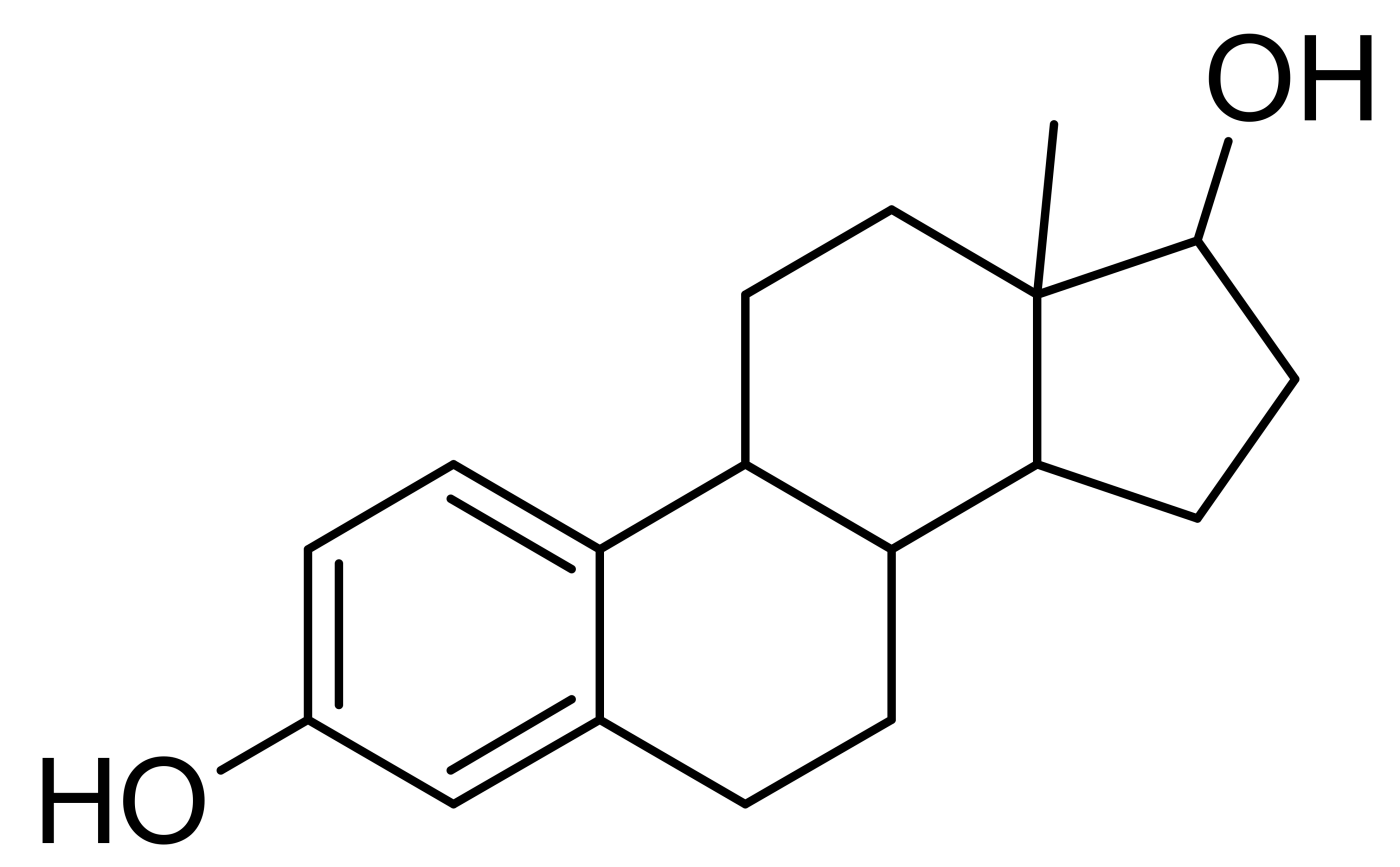 Sigma—Aldrich	50 mM ethanol solution	272.38 g/mol
2-MeOE2	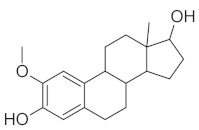 Sigma—Aldrich	10 mM ethanol solution	302.41 g/mol
16α-OHE1	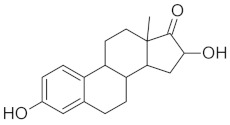 Steraloids	10 mM ethanol solution	286.40 g/mol
Potassium chromate (VI)	K_2_CrO_4_ Sigma-Aldrich	50 mM water solution	194.19 g/mol

## Supplementary Materials

The following are available online, Table S1: IC50 for E2 and K_2_CrO_4_ after simultaneously effect on SKOV-3 cells line, measured by MTT after 24 h and 48 h (expressed in μM); Table S2: IC50 for 2-MeOE2 and K_2_CrO_4_ after simultaneously effect on SKOV-3 cells line, measured by MTT after 24 h and 48 h (expressed in μM); Table S3: IC50 for 16α-OHE1 and K_2_CrO_4_ after simultaneously effect on SKOV-3 cells line, measured by MTT after 24 h and 48 h (expressed in μM); Table S4: IC50 for examined compounds in SKOV-3 cells line after 24 h and 7 days pre-incubation with E2, 2-MeOE2 and 16α-OHE1 and exposure to K_2_CrO_4_, measured by MTT after 24 h and 48 h (expressed in μM).
