# Supplementary material for: Effect of Interaction between 17β-Estradiol, 2-Methoxyestradiol and 16α-Hydroxyestrone with Chromium (VI) on Ovary Cancer Line SKOV-3: Preliminary Study
Source: Molecules. 2020 Nov 9;25(21):5214. doi: 10.3390/molecules25215214 (PMC7665134; doi:10.3390/molecules25215214)
Supplement: Supplementary file 1 [file molecules-25-05214-s001.pdf]

Table S1. IC50 for E2 and K_2CrO_4 after simultaneously effect on SKOV-3 cells line, measured by MTT after 24h and 48h (expressed in µM**)**

E2	K_2CrO_4	0.01 µM E2	0.1 µM E2	10 µM E2	50 µM E2
**24h**	10.9746	10.7894	9.75572	0.0976	1.8907
**48h**	5.4618	4.69393	4.05423	3.11795	6.9395

Table S2. IC50 for 2-MeOE2 and K_2CrO_4 after simultaneously effect on SKOV-3 cells line, measured by MTT after 24h and 48h (expressed in µM**)**

2-MeOE2	K_2CrO_4	0.001 µM 2-MeOE2	0.1 µM 2-MeOE2	10 µM 2-MeOE2	50 µM 2-MeOE2
**24h**	10.9746	12.6869	14.6962	8.62121	12.7909
**48h**	5.4618	6.74551	6.08274	8.85796	0.0903

Table S3. IC50 for 16α-OHE1 and K_2CrO_4 after simultaneously effect on SKOV-3 cells line, measured by MTT after 24h and 48h(expressed in µM**)**

16αHyEstr	K_2CrO_4	0.001 µM16α-OHE1	0.1 µM 16α-OHE1	10 µM 16α-OHE1	50 µM 16α-OHE1
**24h**	10.9746	7.16231	4.15677	8.78224	7.93962
**48h**	5.4618	3.44587	3.17919	2.8159	5.19472

Table S4. IC50 for examined compounds in SKOV-3 cells line after 24h and 7days pre-incubation with E2 , 2-MeOE2 and 16α-OHE1 and exposure to K_2CrO_4, measured by MTT after 24h and 48h(expressed in µM)

E2	K_2CrO_4	K_2CrO_4 + 0.01 µM E2	24h pre-inc. 0.01 µM E2	7 days pre-inc. 0.01 µM E2
**24h**	10.9746	10.7894	21.6823	15.2604
**48h**	5.4618	4.6939	3.38973	1.87134

2-MeOE2	K_2CrO_4	K_2CrO_4 + 0.1 µM 2-MeOE2	24h pre-inc. 0.1 µM 2-MeOE2	7 days pre-inc. 0.1 µM 2-MeOE2
**24h**	10.9746	12.6869	13.8867	10.3379
**48h**	5.4618	6.5521	3.38973	1.87134

16α-OHE1	K_2CrO_4	K_2CrO_4 + 0.001 µM 16α-OHE1	24h pre-inc. 0.001 µM 16α-OHE1	7 days pre-inc. 0.001 µM 16α-OHE1
**24h**	10.9746	7.16231	6.97628	35.9269
**48h**	5.4618	3.44587	2.51319	2.23566
